# Complete polarization of electronic spins in OLEDs

**DOI:** 10.1038/s41467-021-22191-3

**Published:** 2021-04-06

**Authors:** Tobias Scharff, Wolfram Ratzke, Jonas Zipfel, Philippe Klemm, Sebastian Bange, John M. Lupton

**Affiliations:** Institut für Experimentelle und Angewandte Physik, Universität Regensburg, Regensburg, Germany

**Keywords:** Electronic devices, Semiconductors

## Abstract

At low temperatures and high magnetic fields, electron and hole spins in an organic light-emitting diode become polarized so that recombination preferentially forms molecular triplet excited-state species. For low device currents, magnetoelectroluminescence perfectly follows Boltzmann activation, implying a virtually complete polarization outcome. As the current increases, the magnetoelectroluminescence effect is reduced because spin polarization is suppressed by the reduction in carrier residence time within the device. Under these conditions, an additional field-dependent process affecting the spin-dependent recombination emerges, possibly related to the build-up of triplet excitons and their interaction with free charge carriers. Suppression of the EL alone does not prove electronic spin polarization. We therefore probe changes in the spin statistics of recombination directly in a dual singlet-triplet emitting material, which shows a concomitant rise in phosphorescence intensity as fluorescence is suppressed. Finite spin-orbit coupling in these materials gives rise to a microscopic distribution in effective g-factors of electrons and holes, Δg, i.e., a distribution in Larmor frequencies. This Δg effect in the pair, which mixes singlet and triplet, further suppresses singlet-exciton formation at high fields in addition to thermal spin polarization of the individual carriers.

## Abstract (web summary)

Though literature reports magnetoelectroluminescence (MEL) affects in organic light‐emitting diodes (OLEDs), probing the organic layer’s effective spin polarization remains a challenge. Here, the authors utilize dual singlet‐triplet emitting OLEDs to reveal the spin polarization in the materials.

## Introduction

A century ago, the experiment of Stern and Gerlach arguably led to one of the most important, exciting, and unanticipated discoveries of physics^1^. While trying to measure the magnetic moment of atoms associated with the orbital angular momentum of electrons, the intrinsic magnetic moment of the electron was inadvertently discovered—the electron “spin”, a property, which Dirac subsequently proved to be a direct consequence of the relativistic formulation of Schrödinger’s matter-wave equation. Manipulation of the electron spin as inspired by the breakthrough of Stern and Gerlach has long been commonplace in “spintronics” technology^2^, in magnetic-resonance imaging^3^, and in quantum-information science^4^; it is even used in high-energy physics in studies of spin-dependent scattering processes^5^. But the spin of an electron is also central to the operational principles of seemingly disparate processes: in an organic light-emitting diode (OLED), for example, electrons and holes recombine in a spin-dependent fashion to generate light. Since the recombinant singlet and triplet molecular excited-state species are non-degenerate, the statistical distribution of the spin permutation symmetries of the recombination products determines the overall power efficiency of a device^6^. Similar phenomenology appears at the heart of photochemical reactions triggered by photoinduced electron transfer^7^. It has, for example, been proposed that retinal pigment-protein complexes of some bird species support the formation of spatially separated spin-correlated carrier-pair states, the singlet-triplet recombination yield of which can be influenced by the changes in spin precession arising on magnetic-field scales as small as geomagnetic-field strengths^8^. These processes depend on the permutation symmetry of a spin pair, and not on a Stern-Gerlach type of spin polarization.

OLEDs offer a potentially interesting semiconductor basis to investigate spin polarization phenomena because spin-orbit coupling (SOC) effects are generally weak in these materials^9–14^. The intrinsic magnetic moment and angular momentum of a particle such as an electron is commonly stated as the dimensionless magnetic moment, the g-factor, which relates a measured magnetic moment to the particle’s angular-momentum quantum number and a quantum of angular momentum like the Bohr magneton *μ*_B_. For a free, i.e., unbound, electron, the spin g-factor is 2.002319 to the first six significant digits, slightly off from the reciprocal of the spin quantum number. This famous anomalous magnetic moment of the electron is a consequence of relativistic quantum electrodynamics and the higher-order corrections, i.e., electron-photon interaction loops, to the vertex functions describing the coupling of electromagnetic radiation and matter. While this definition sounds complicated, it is easy to rationalize by an electron spin in a magnetic field. Depending on whether the electron spin is aligned in parallel or antiparallel to the field axis, an energy difference arises corresponding to *gμ*_B_*B*. The effective g-factor can therefore simply be measured by the electron paramagnetic resonance condition, the absorption of electromagnetic radiation of a frequency *hν* = *gμ*_B_*B*. In organic semiconductors, both electron and hole spin behave rather like free electrons in terms of their g-factors, with very subtle shifts arising from the combination of spin and angular momentum, i.e., SOC^14^. This definition of the effective g-factor by the magnetic-resonance condition is particularly helpful because it implicitly includes the effect of spin-spin (dipolar or exchange) interactions, i.e., the zero-field splitting of the electron-hole pair, as well as electron-nuclear hyperfine coupling^15^.

OLED-like devices with ferromagnetic electrodes exhibit a range of intriguing magnetoresistance and magnetoelectroluminescence (MEL) phenomena, which suggest that it may be possible to inject spin-polarized currents into organic semiconductors^16,17^. However, it is very challenging to prove with certainty that a net magnetization is actually established within the organic semiconductor since it has not been possible to apply conventional techniques of spin spectroscopy such as Kerr-rotation and Faraday-rotation^18,19^. Spin-polarized photoelectron spectroscopy may be sensitive to the interface of the ferromagnetic electrode and the semiconductor^20^, whereas muon scattering provides a somewhat indirect measure of electronic spin polarization and requires major research infrastructure^21^.

A conceivable way to examine the emergence of effective spin polarization is through the substantial change in spin statistics of the recombinant electron-hole pair, i.e., the change in the ratio of molecular singlet to triplet excitations. However, OLED structures with potentially spin-polarizing ferromagnetic electrodes show only minimal relative changes in intensity, of the order of a few percent^22,23^. More importantly, most OLED materials emit only from either the singlet or the triplet state, but not from both. It is therefore not possible to conclusively assign the loss or gain in intensity within one recombination channel with certainty to a change in spin statistics alone. The most compelling demonstration to date of spin polarization occurring in an OLED structure reported suppression of EL intensity by almost 50% due to thermal spin polarization (TSP)^24^. When the Zeeman energy of a spin in a magnetic field oriented along the *z*-axis becomes comparable to the thermal energy *k*_B_*T*, the product of Boltzmann’s constant and temperature, the probability of spins polarized along ±_±_*z* at thermal equilibrium becomes a function of temperature and magnetic field,

1 $${P_ \uparrow ^{{\mathrm{eq}}} = \frac{1}{{1 + \exp \left( {\frac{{g\mu _{\mathrm{B}}{\mathrm{B}}}}{{k_{\mathrm{B}}T}}} \right)}}}\quad{\mathrm{and}}\quad P_ \downarrow ^{{\mathrm{eq}}} = 1 - P_ \uparrow ^{{\mathrm{eq}}}.$$

Unpolarized injected spins equilibrate due to the spin-lattice relaxation within a time *τ*_*s*_ to a distribution given by Eq. (1). However, the lifetime of the free spins in the organic semiconductor is limited by the formation of the strongly bound excitons, so that the resulting spin-statistics is the composition of unpolarized and polarized spins. The degree of mixture of both is then defined by the relaxation time *τ*_*s*_ and the time available for relaxation, the free-carrier lifetime *τ*_*c*_. Equation (1) must therefore be modified to

2 $${P_ \uparrow = \left( {\frac{1}{2} - P_ \uparrow ^{{\mathrm{eq}}}} \right)\exp \left( { - \frac{{\tau _{\mathrm{c}}}}{{\tau _{\mathrm{s}}}}} \right) + P_ \uparrow ^{{\mathrm{eq}}}.}$$

Spins tend to align in parallel to the external magnetic field, so that recombination preferably arises in the triplet state. In a material which exhibits only fluorescence from the singlet and no phosphorescence from the triplet state, the overall EL yield, i.e., the probability of light emission from singlets, is given by

3 $$\begin{array}{*{20}{c}} {P_{\mathrm{S}} = P_ \uparrow \cdot P_ \downarrow = P_ \uparrow - P_ \uparrow ^2.} \end{array}$$

This probability drops with increasing magnetic field following the Boltzmann activation. While Wang et al. indeed showed suppression of EL intensity with the magnetic field^24^, the form of the functional field dependence left room for interpretation. More importantly, suppression of EL intensity alone does not, strictly, prove spin polarization—to do this, an anticorrelation must be demonstrated between the reduction in the yield of singlets and the increase in triplet yield.

Here, we demonstrate a complete suppression of the EL by strong magnetic fields at low temperatures in a fluorescent OLED material. The degree of EL suppression depends on temperature, but also on current, with the effect of the apparent spin polarization vanishing with increasing temperature and current. We introduce a dual singlet-triplet emitting compound^25–27^, which shows a direct anticorrelation between the suppression of singlet fluorescence and the increase of triplet phosphorescence due to the formation of a spin-polarized ensemble of electron-hole pairs. The approach offers direct visualization of the Stern-Gerlach-type of spin polarization in OLEDs—a spectroscopic analog of space quantization, and reveals subtle SOC effects in these low-atomic-order-number materials. TSP is shown to be superimposed by SOC-related spin-mixing effects, which are responsible for a microscopic distribution in effective Larmor frequencies of the spins, i.e., in effective g-factors. When the effect of TSP is suppressed at high currents and temperatures, this Δg effect^28–35^ becomes apparent as a further mixing channel between singlet-type and triplet-type spin pairs. OLEDs, therefore, offer an approach to differentiate between changes in spin-dependent recombination due to single-carrier spin polarization effects^36^ and spin-pair correlation effects^37^.

## Results

### Singlet emitters

**Fig. 1:**
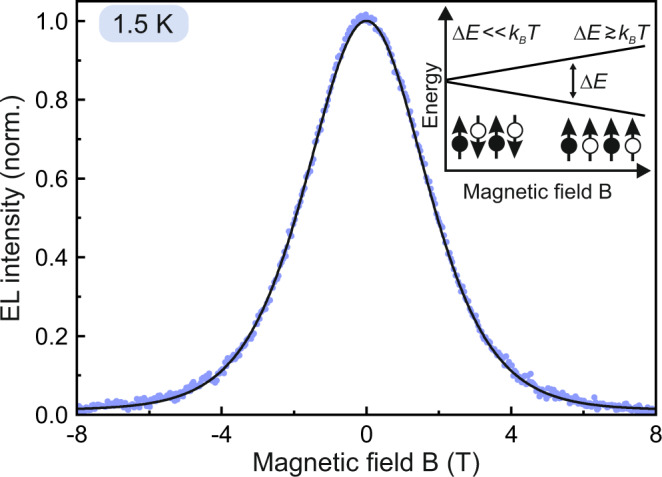
Thermal spin polarization (TSP) in a “super-yellow” poly(phenylene-vinylene) (SYPPV) OLED at 1.5 K. The magnetic field splits the energy of the spins with respect to their orientation in the field. Once this splitting exceeds the thermal energy *k*_B_*T*, spins can become polarized so that electron and hole pairs recombine preferentially into the triplet excited-state manifold of the molecule, which generally does not emit light. The electroluminescence (EL) intensity of the OLED is recorded as a function of magnetic field strength. The change in EL intensity with the magnetic field, the magnetoelectroluminescence (MEL), follows Boltzmann activation of the singlet-triplet ratio [black line, Eq. (3)], demonstrating a degree of polarization of over 99% at ±8 T.

Although there are several reports of OLED magnetoresistance^32^ and photoconductivity^38^ at high magnetic fields, given the experimental challenges involved, there are only two prior studies of MEL under such conditions^24,39^. To accurately measure the EL intensity at very low currents and brightnesses requires the use of a single-photon counter which is not affected by stray magnetic fields of the cryostat. Photomultipliers and other optical intensifiers cannot be used for this challenge as the Lorentz force exerted by the stray fields induces substantial artifacts in the detector sensitivity. Point detectors such as avalanche photodiodes are also of limited use since the OLED emits over a pixel surface area and any elastic deformation of the device, sample holder or cryostat due to magnetic forces will induce artefacts in the measurement which cannot be accounted for. Instead, we use a single-photon counting area detector, a scientific CMOS (sCMOS) camera^27^, which offers a detection quantum efficiency of almost 80% together with the ability to correct for any unwanted mechanical movement, provided that the image of the OLED pixel projected onto the camera is smaller than the area of the camera chip. Figure 1 shows the change in EL intensity of an OLED made of the commercial poly(phenylene-vinylene) (PPV) derivative “super-yellow PPV” (SYPPV), operated in constant-current mode, at a temperature of 1.5 K and a very low current of 550 nA, corresponding to a voltage of around 5.5 V. The sketch illustrates how TSP arises as the Zeeman splitting of spin states becomes comparable to the thermal energy k_B_*T*. The entire measurement takes 4 h for two complete sweeps up and down in the magnetic field, and the curve shows the average of both sweep directions. The functionality perfectly follows the prediction of Eq. (3), with the only free fitting parameter being the ratio of spin residence time in the field to spin-lattice relaxation time, *τ*_*c*_/*τ*_*s*_ = 5.6. The fit suggests that a spin polarization of >99% can be reached. In contrast to the report of Wang et al.^24^, it was not possible to extract spin residence and relaxation times directly from transient EL measurements. Wang et al.^24^ appeared to observe an additional slow relaxation process at high magnetic fields which they attributed to the TSP mechanism. However, the transient EL measured at low currents displayed in Supplementary Fig. S1 and discussed in Supplementary Note 1 shows clearly that the magnetic field actually quenches the fluorescence immediately, i.e., there are no resolvable EL dynamics associated with TSP; no additional relaxation processes appear at high fields. This absence of additional dynamics implies that the observed transient EL originates from spin-polarized charge carriers, i.e., the transient dynamics bear no relation to spin-lattice relaxation.

**Fig. 2:**
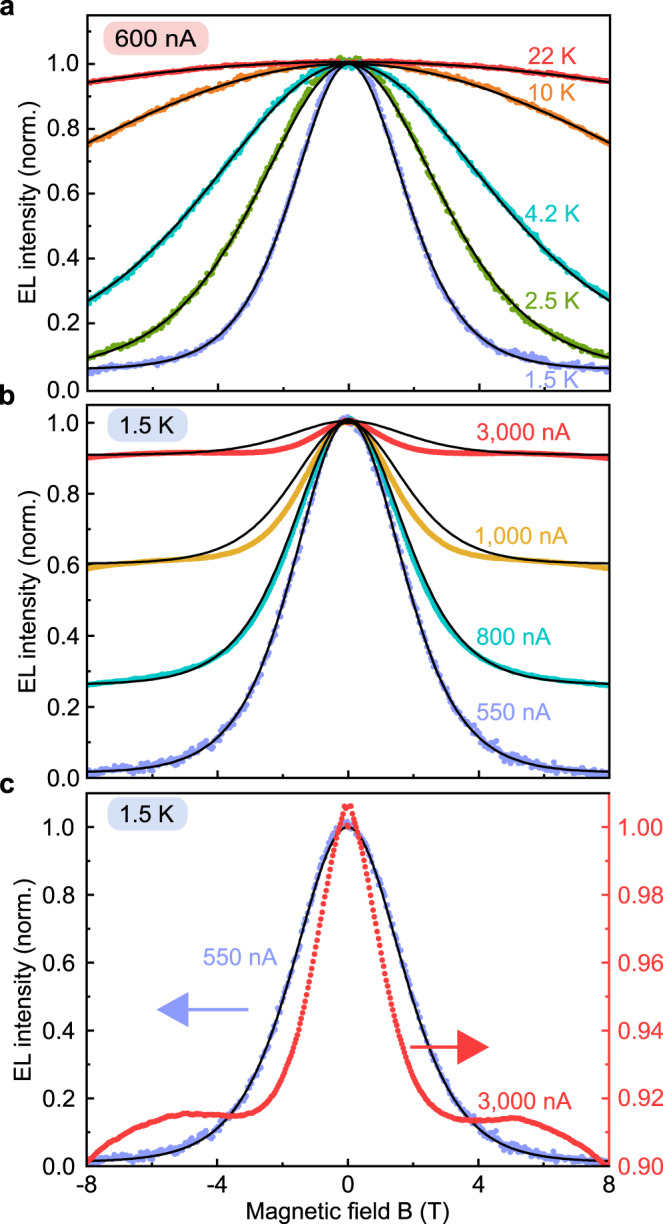
Current and temperature dependence of TSP-induced MEL of a SYPPV OLED. **a** Temperature dependence at a device current of 600 nA. At elevated temperatures, the functionality of the MEL curves is in good agreement with the Boltzmann-type activation expected of TSP. **b** Current dependence at a temperature of 1.5 K. At higher currents, the functionality narrows with respect to the Boltzmann activation behavior [black lines, following Eq. (3)], most likely because long-lived triplet excitons are quenched by free polarons to form additional singlets. **c** Direct comparison of MEL curves at 550 nA and 3000 nA drive current. Note the different scales for the blue and red curves.

Next, in Fig. 2a, we probe TSP by the temperature dependence of the OLED MEL. As expected, an increase of the temperature results in a weaker polarization of the spins and therefore in a reduced suppression of the EL at strong magnetic fields^24^. The black lines show the predicted functionalities from Eq. (3), which are in excellent agreement with the measurements using one common *τ*_*c*_/*τ*_*s*_ value of 3.7 for all datasets. The reason these experiments have to be performed at such low currents is apparent from the current dependence of TSP shown in Fig. 2b. As the current increases, the overall magnitude of the MEL effect is reduced^24^, suggesting that the TSP is quenched. For TSP to occur, carriers must reside in the device for a sufficiently long time to be able to equilibrate in the field by spin-lattice relaxation^24^. The higher the device current, the shorter the carrier residence time and the greater the probability of scattering between two carrier spins becomes. This latter effect will tend to randomize the spin orientations again within the device^24^. In addition, the shape of the functionality of the MEL curve changes with increasing current, as seen in the direct comparison of two curves in Fig. 2c: MEL can no longer be described accurately by Eq. (3). It is known that triplet excitons can interact with free charge carriers or with each other to annihilate and generate additional singlet states, thereby forming a singlet population that exceeds the spin-statistical limit^40,41^. An external magnetic field tends to inhibit these processes, thereby reducing the overall number of singlets formed, in addition to the TSP singlet-suppression effect^42^.

To test for the possible influence of such delayed fluorescence from triplet-triplet annihilation, we examined time-resolved EL of the afterglow of the OLED, i.e., the turn-off behavior, as shown in Supplementary Fig. S2. We observe only a sharp EL overshoot at turn-off, followed by a weak afterglow over a few microseconds, which is unaffected by temperature, magnetic field, and voltage. By applying a negative or positive bias offset to the driving pulse, the afterglow can be either accelerated or decelerated—a clear indication of the release of trapped or accumulated charge carriers in the device^43–50^. In contrast, time-resolved measurements of the turn-on behavior, shown in Supplementary Fig. S3, indicate the emergence of an additional rise in EL intensity at high drive currents. In apparent disagreement with the earlier observations of Wang et al.^24^, these features are sensitive to temperature and magnetic field. However, as would be expected, the field dependence vanishes for temperatures above 50 K, indicating that TSP is only relevant at low temperatures where the Zeeman splitting energy exceeds the thermal energy. The sharp EL overshoot at turn-on suggests an accumulation of charge carriers at internal energy barriers^43–51^. From these observations, we conclude that there is no significant delayed fluorescence from triplet-triplet annihilation in our devices, but instead a substantial effect from accumulated or trapped charge carriers arises. These carriers of limited mobility can be fully polarized by the magnetic field and can also interact with the long-lived triplet excitonic states^52^. The pronounced change of MEL curvature at high currents, leading to a strong deviation from Eq. (3), is therefore likely a signature of triplet-exciton polaron quenching^53^ with accumulated spin-polarized polarons.

### Dual singlet-triplet emitters

**Fig. 3:**
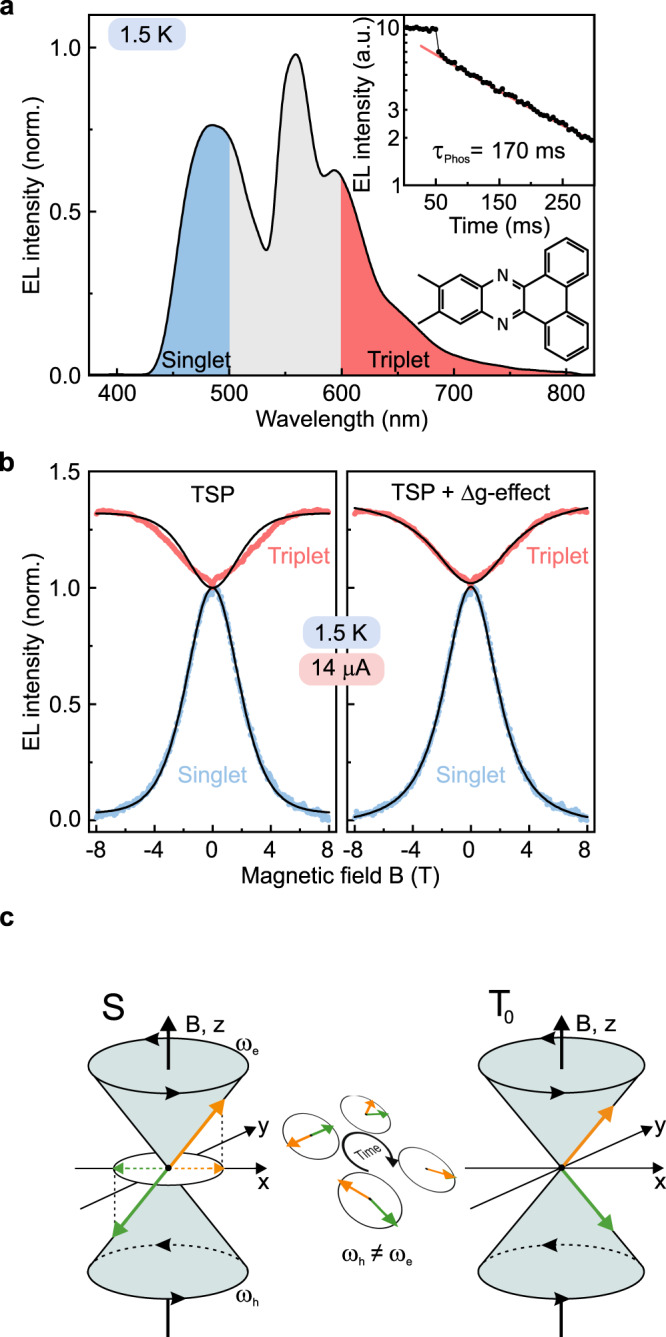
MEL of dual singlet-triplet emitting OLEDs, resolving a suppression of singlet fluorescence with a concomitant rise in triplet phosphorescence. **a** EL spectrum of an OLED with the dual emitter DMDB-PZ co-evaporated with CBP, at 1.5 K. The shaded regions indicate the spectral range selected by filters to distinguish between fluorescence (blue) and phosphorescence (red). The inset shows a transient EL measurement, revealing a triplet phosphorescence lifetime of 170 ms. **b** Change of singlet and triplet luminescence intensity at 1.5 K under a device current of 14 µA, with a fit of the Boltzmann-TSP function [Eq. (3)] and a fit of the TSP functionality modified for the Δg effect [Eq. (4)]. **c** Sketch of the singlet-triplet mixing process in the pair due to the Δg effect.

Suppression of the singlet yield alone does not imply the generation of spin-polarized carriers in the OLED: conversion of singlet pairs to superposition-state triplet pairs suppresses fluorescence but does not lead to spin polarization. A spectroscopic test of signatures of the formation of polarized spins requires a comparison of the populations of singlet and triplet excitons and an examination of the functionalities of the dependence on magnetic-field strength. To do this, we introduce a surprisingly simple OLED emitter with intrinsic dual singlet-triplet emission because of the SOC arising from the mixing of π-orbitals with the non-bonding orbitals of a phenazine moiety^25–27^. The structure of 11,12-dimethyldibenzo[a,c]phenazine (DMDB-PZ) is shown in Fig. 3a. The material is coevaporated with 4,40-bis(N-carbazolyl)-1,10-biphenyl (CBP) to yield an OLED with distinct dual singlet and triplet emission, split by approximately 420 meV. A representative EL spectrum, recorded at a temperature of 1.5 K, is plotted in Fig. 3a. The inset shows a transient EL measurement, which demonstrates a triplet phosphorescence lifetime *τ*_phos_ of 170 ms at 1.5 K. Unlike conventional organometallic triplet emitters^6,54^, SOC is comparatively weak in these compounds, so that the singlet population is only partially quenched by intersystem crossing (ISC) to the triplet manifold. Because singlet and triplet are highly non-degenerate, the ratio of singlet to triplet emission intensity is not affected by temperature as in thermally activated delayed fluorescence^55–57^. Also, since every single chromophore shows dual emission, the detected singlet-triplet ratio in EL does not depend on triplet diffusion to an emitting site. Unlike dual-emitting conjugated polymers previously used to probe the evolution of the singlet-triplet ratio in MEL^58^, the ratio of fluorescence to phosphorescence is therefore not inherently temperature dependent^59^. To measure the spectrally resolved MEL effect of these dual-emitting OLEDs requires a substantial modification to the optical imaging setup. Filters are used to select the spectral regions marked in the spectrum in Fig. 3a, allowing changes in EL intensity to be resolved down to levels of 1000 ppm. Figure 3b shows the evolution of singlet and triplet EL intensity with the magnetic field, at a temperature of 1.5 K and for a device current of 14 μA. The fluorescence is quenched by up to 98%, but the maximal simultaneous increase in triplet intensity is only 34%. The main reason for this discrepancy lies in the spin statistics of electron-hole recombination, which occurs following a ratio of 1:3 of singlets to triplets. Complete depletion of the singlets by converting the pairs to triplets will quench the singlet yield by 100%, but will only raise the triplet yield by at most 1/3. Although it is also conceivable that the overall radiative phosphorescence yield of the dual emitters changes with magnetic field strength since the energetic separation between sublevels of the molecular triplet states will change, the experimental observation suggests that the field dependence of the phosphorescence intensity is primarily due to spin statistics.

### Role of spin-orbit coupling

Neither fluorescence nor phosphorescence can be described purely by the Boltzmann-type TSP functionality of Eq. (3) (Fig. 3b, left), indicating the presence of a further spin-mixing process in addition to TSP. Since spin-mixing processes related to hyperfine interactions, exchange interactions and the zero-field splitting occur at much smaller magnetic-field strengths, i.e., below hundreds of millitesla^38^, we attribute this second mechanism to the “Δg effect” illustrated in Fig. 3c. Because of the weak but finite SOC in these materials^14^, a distribution in effective g-factors of electrons and holes exists within a carrier pair, implying that the carriers precess at slightly different frequencies in an external magnetic field. For small fields, spin precession is dominated by the local disorder in effective magnetic fields, which arises due to the distribution of nuclear magnetic moments, i.e., the hyperfine coupling^60^. At high fields, SOC effects dominate the precession frequency: spins in singlet carrier pairs $\left| { \uparrow \downarrow } \right\rangle - \left| { \downarrow \uparrow } \right\rangle$ precess to form superposition-state triplet pairs $\left| { \uparrow \downarrow } \right\rangle + \left| { \downarrow \uparrow } \right\rangle$^30^.

To a first approximation, the suppression of the yield of singlet excitons due to the Δg-mechanism in the high-field regime has been described by a phenomenological Lorentzian functionality^38,61^, which is rationalized simply in terms of the damping over time of the coherent spin mixing between singlet and triplet configurations of the carrier pair. Adding such functionality to Eq. (3) gives a phenomenological expression^32^ of the form

4 $$\begin{array}{*{20}{c}} {P_{\mathrm{S}} = P_ \uparrow - P_ \uparrow ^2 - \alpha \frac{{B^2}}{{{\mathrm{{\Delta}}}B_{1/2}^2 + B^2}} + d} \end{array}$$

for the singlet yield, which shows remarkably good agreement with the measurement on the righthand side of Fig. 3b. The fitting parameters used involve only the ratio of spin residence to relaxation times, *τ*_*c*_/*τ*_*s*_, an effective amplitude *α* for the Δg effect, and a width of the Lorentzian ${\Delta}B_{1/2}$, which relates to the difference in precession frequencies of electron and hole within the pair. The fitting procedure, described in the Supplementary Note 2, does not include data points below ±800 mT and only accounts for the effective relative contribution of these low-field effects by an offset *d*: the precession frequency of Δg-mixing is determined solely by SOC at high fields, whereas spin mixing due to the hyperfine fields dominates at low to intermediate fields^61^.

For the MEL in the phosphorescence channel, i.e., the OLED triplet formation yield, the modification in Eq. (4) improves the fit compared to the pure TSP functionality of Eq. (3), but slight discrepancies with regards to the experimental data remain. Considering the very long triplet excited-state lifetime and the possibility of generating additional triplet states due to TSP and the Δg effect, a substantial accumulation of triplet excitations is expected to occur in a device. This accumulation will result in a higher quenching probability of triplets by either triplet-triplet annihilation or by triplet-polaron interactions^53^ since the overall triplet population increases at large magnetic fields. These additional dynamical processes are not considered in this simple static model. We stress that the fit function is entirely qualitative in nature and cannot be used to extract effective SOC strengths of the material since the fit to the Δg effect is not sufficiently sensitive to Δ*B*_1/2_ over the limited range of magnetic fields probed in the experiment^32^. It serves predominantly to extract the degree of TSP from the MEL. To determine Δg precisely^31^, high-field electron paramagnetic resonance spectroscopy is necessary, i.e., EPR measurements at static fields where hyperfine-induced broadening of the resonance no longer dominates the resonance spectrum as recently demonstrated in the context of OLEDs^14,32,62^.

**Fig. 4:**
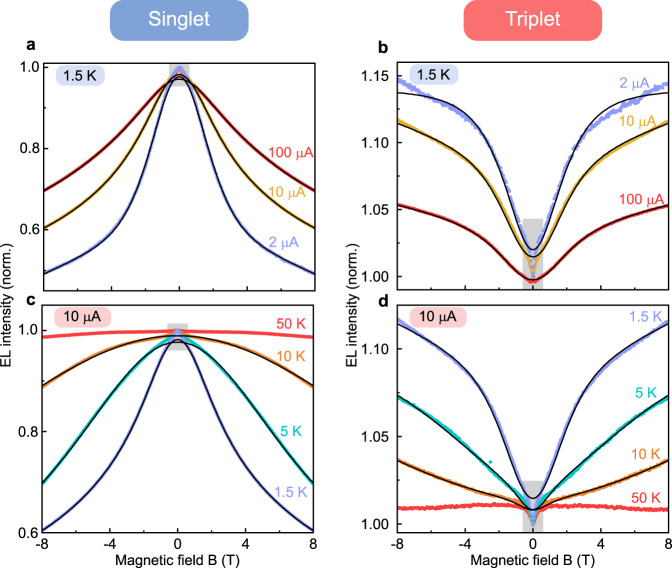
Current and temperature dependence of the singlet and triplet EL intensity of a dual-emitting OLED. The current dependence (**a**, **b**) is shown at a temperature of 1.5 K and the temperature dependence (**c**, **d**) at a current of 10 μA. The lines show a fit to the data of the combined functional dependence of TSP and the Δg effect following Eq. (4). At higher currents and at low temperatures, a narrow feature is observed around zero field (gray areas), which is attributed to the effect of spin mixing due to hyperfine interactions. This gray region is excluded in the fitting procedure. The triplet intensity is quenched at high fields for the 50 K curves at 10 μA, so there is no meaningful fit possible.

Resolving this direct anticorrelation between the intensities of fluorescence and phosphorescence due to TSP requires careful optimization of the device structure and operating conditions to maximize the residence time of uncorrelated free charge carriers in the external magnetic field. In some device structures, the carriers may become trapped at the interface between two layers. While this trapping raises the effectiveness of TSP, it can also enable direct injection into the triplet state^63^, so that the ratio of singlet to triplet emission does not perfectly reflect the equilibrium spin statistics. However, changes in spin statistics will still be captured by changes in the fluorescence-to-phosphorescence ratio. Figure 4 illustrates how an increase in temperature or device current has a dramatic effect on the MEL functionality, which can appear to be quite distinct in the singlet and triplet channels. As in the case of SYPPV OLEDs (Figs. 1, 2), as the device current at a temperature of 1.5 K increases in Fig. 4a, b, the overall amplitude of the MEL effect decreases. The same trend is observed by increasing the temperature in Fig. 4 c, d. In both cases, the shape of the curve appears to broaden - with increasing temperature or current, respectively -  because of the increased relative contribution of the Δg effect to the MEL as indicated by the fit of Eq. (4) to the data. These devices also show a sharp MEL feature around zero field, which presumably results from the spin-mixing process arising from electron and hole spin precession within the hyperfine fields and is not accounted for with the simple model used here^58^. Interestingly, this feature appears sharper and more pronounced in the increase of phosphorescence intensity with the magnetic field than in the suppression of the fluorescence. This observation on the scale of several hundred mT is in contrast with measurements on similar diodes in the range of a few mT^27^, which show a quantitative anticorrelation between singlet and triplet and the opposite effect with a rise in singlet yield and a suppression of triplets. At fixed temperatures, as the current is lowered, the hyperfine-field feature in the triplet channel appears to become sharper. As the current in panel b is raised to 100 μA, the functionality even inverts, so that the slope of the MEL of singlet and triplet have the same sign, i.e., the two intensities are no longer anticorrelated in contrast to the conventional situation^27^. This inversion indicates a sensitive interplay between effective carrier lifetime, which is related to the device current, and the hyperfine-induced spin mixing^27^, in addition to possible level-crossing effects in the triplet exciton at intermediate field strengths, which complicates the MEL functionality substantially at fields below those where TSP becomes significant. Although, as discussed in Supplementary Note 3, in principle, experiments using contact-gas cooling can be affected by Joule heating at large bias currents, the effects discussed here prevail even when the sample is fully immersed in liquid helium implying that Joule heating effects are compensated even under conditions of contact-gas cooling for the drive currents employed.

## Discussion

TSP is a rather subtle effect, which is easily quenched under strong currents. However, the fact that TSP can be observed at near unity polarization implies that electron paramagnetic resonance signals should become huge at high fields and very low temperatures. In the dual emitters, we expect this effect to become so strong that illumination of the device with THz radiation resonant with the magnetic-dipole transitions of the Zeeman-split spin states will visibly alter the singlet-triplet ratio and hence the emission color of the OLED. Pulsed magnetic resonance experiments should then provide unique means to differentiate between coherent and incoherent spin-mixing processes, revealing how spins in organic semiconductors interact with each other^15^ and with the environment. An intriguing question is whether the dramatic electronic spin polarization can be transferred to the nuclear spin bath as, for example, in silicon devices^64^ or in anthracene crystals^65^. Hyperfine interactions may lead to hyperpolarization of the nuclei, which would alter the low-field magnetoresistance and MEL response. At present, there is no clear route to transferring the device swiftly from the high-field to the low-field regime given the inherent inertia in magnetic-field sweeps of superconducting magnets. Alternatively, nuclear magnetic resonance should also have an appreciable effect on EL under these conditions, providing complimentary access to the hyperfine interaction to probe for nuclear spin polarization^60^. We expect that the conclusive observation of near-perfect spin polarization in OLEDs will offer further insight into the properties of electron spins in aromatic hydrocarbons, and may help explain the puzzling decoupling of spin and charge transport in these materials^66^, which appears to be responsible for some of the magnetoelectronic effects reported previously.

## Methods

### OLED fabrication

Glass substrates covered with 100 nm of indium-tin oxide (ITO) were obtained from Präzisions Glas & Optik (Germany) and structured by etching the ITO in FeCl_3_/HCl solution. Next, the surface was treated in an ultrasonic cleaner in successive baths of acetone, 2% Hellmanex III solution (Hellma Analytics), and isopropanol. After each cleaning step, the substrates were flushed with ultrapure water. Finally, they were treated by oxygen plasma cleaning (40 kHz, 80 W, plasma technology GmbH) for 30 min followed by UV/ozone exposure (Novascan Technologies) on a hot plate at 100 °C for a further 30 min. Directly after cleaning, an 80 nm thick hole-injector layer of poly(*3,4*-ethylenedioxythiophene) polystyrene sulfonate (PEDOT:PSS; Clevios P VP AI 4083, Heraeus) was spin-coated on the ITO surface, and the substrates were transferred to a nitrogen glovebox where they were baked out on a 150 °C hot plate for 30 min. For the emissive layer of the fluorescent SYPPV devices, 5 mg_·_ml^−1^ of SYPPV was dissolved in toluene and spin-coated to give a thickness of ~100 nm. For the top electrodes, barium (3 nm) and aluminum (250 nm) layers were deposited by thermal evaporation through a shadow mask, yielding an active pixel area of 3 mm^2^. The dual-emitting devices were produced in a similar fashion except that the emitting layer was deposited by thermal evaporation. For the emissive layer, 4,40-bis (N-carbazolyl)-1,10-biphenyl (CBP, Ossila Ltd.) and 11,12-dimethyldibenzo[a,c]phenazine (DMDB-PZ, Sigma Aldrich) were deposited by thermal co-evaporation at a ratio of 97:3 to yield a thickness of 60 nm. All devices were encapsulated with a 500 nm thick layer of N,N′-bis(3-methylphenyl)-N,N′-diphenylbenzidine (TPD, Ossila Ltd.) by thermal evaporation to protect from air and to reduce thermal stress during cooling.

### Magnetic-field setup

The samples were placed in a cryostat with a split-coil superconducting magnet and windows for optical access (American Magnetics). For the fluorescent devices, the EL emission was directly projected onto an sCMOS camera (Hamamatsu) using a lens system with the detector placed roughly 0.7 m away from the magnet. For the dual-emitting devices, an additional optical unit was positioned in the beam path, which allowed the spectral separation of fluorescence and phosphorescence. First, the incident light impinges on a dichroic mirror, which splits the beam at 532 nm. To achieve further spectral separation, a 500 nm short-pass filter and a 600 nm long-pass filter, respectively, were included in the beam paths. Both beams were focused as non-overlapping images onto the same sCMOS sensor, which allows a parallel and synchronous detection of fluorescence and phosphorescence intensity. Alternatively, the EL emission could be focused on a spectrometer. For detection, we used a gated iCCD camera (Andor iStar 720), allowing for time and spectrally resolved EL measurements. The OLEDs were operated in constant-current mode using a low-noise source-measure unit (Keithley 2400). During the measurement, the device voltage was recorded by an additional dc digital multimeter (Keysight 34470 A). The sweep rates of the magnet were 5 mT_·_s^−1^ for the range 0 T < |B| ≤ 6.4 T and 4 mT_·_s^−1^ for 6.4 T < |B| ≤ 8 T. The magnetic field was applied in a plane perpendicular to the sample surface. The cryostat comprises a variable temperature insert (VTI) which allows temperatures between 1.5 K and room temperature to be reached.

### Fitting procedures

Details of the fitting parameters, following the fitting procedure described in Supplementary Note 2, are given in the Supplementary Information Tables S1–S4.

## Supplementary information

Supplementary Information

## Data Availability

The raw data that support the plots within this paper and the other findings of this study are available from the corresponding author upon reasonable request.
