# Supplementary material for: Complete polarization of electronic spins in OLEDs
Source: Nat Commun. 2021 Apr 6;12:2071. doi: 10.1038/s41467-021-22191-3 (PMC8024367; doi:10.1038/s41467-021-22191-3)
Supplement: Supplementary file 1 — Supplementary Information [file 41467_2021_22191_MOESM1_ESM.pdf]

Supplementary Information

Complete polarization of electronic spins in OLEDs

T. Scharff, W. Ratzke, J. Zipfel, P. Klemm, S. Bange, and J. M. Lupton

Institut für Experimentelle und Angewandte Physik, Universität Regensburg, Universitätsstraße 31, 93053 Regensburg, Germany

Supplementary Notes

Supplementary Notes 1: Time-resolved EL measurements

Time-resolved EL was measured by applying voltage pulses of 20 ms duration using an Agilent Technologies 8114A pulse generator and an avalanche photodiode (APD, PerkinElmer SPCM-AQR-13) with an event counter (Nanoharp 250, Picoquant GmbH) as the detector.

Supplementary Note 2: Fitting functions and parameters

As noted in the main text, the thermal equilibrium value of spin-up and spin-down particles is determined by the Boltzmann distribution,

$$P_{\uparrow}^{\mathrm{eq}} = \frac{1}{1+\exp\left(\frac{g\mu_{\mathrm{B}}B}{k_{\mathrm{B}}T}\right)}$$

and

$$P_{\downarrow}^{\mathrm{eq}} = 1 - P_{\uparrow}^{\mathrm{eq}}, \qquad (1)$$

with g the electron g-factor, μ_B the Bohr magneton and k_B Boltzmann's constant. Taking into consideration a spin relaxation time τ_s and an effective free-carrier residence time τ_c, the fraction of spin-polarised charge carriers at recombination is given by

$$P_\uparrow = \left(0.5 - P_\uparrow^{eq}\right) \exp\left(-\frac{\tau_c}{\tau_s}\right) + P_\uparrow^{eq}. \quad (2)$$

With these considerations, one obtains the following formation probabilities of singlet and triplet states[1], namely

$$P_S = P_\uparrow \cdot P_\downarrow = P_\uparrow - P_\uparrow^2 \quad (3)$$

and

$$P_T = 1 - P_S\,. \quad (4)$$

For the pure MEL response due to TSP, the following fit functions were used

$$\mathrm{MEL}_S^{TSP}(B) = \frac{P_S(B)}{P_S(0)} = 4 \cdot \left(P_\uparrow - P_\uparrow^2\right) \quad (5)$$

$$\mathrm{MEL}_T^{TSP}(B) = \frac{P_T(B)}{P_T(0)} = \frac{4}{3} \cdot (1 - P_S) \quad (6)$$

with the assumption that, at zero field, singlet and triplet states are formed in a ratio of 1:3. We note that this assumption is approximate because hyperfine fields can give rise to weak spin mixing, raising the singlet yield at zero field [3]. The only free fitting parameter is the ratio τ_c/τ_s.

To account for the Δg-mixing mechanism, a Lorentzian functionality of the form

$$\alpha \frac{B^2}{\Delta B_{1/2}^2 + B^2}$$

is added to the yields of singlet and triplet excitons, where α defines the relative strength of the effect compared to TSP and $\Delta B_{1/2}$ is the width of the Lorentzian that reflects the difference in precession frequencies of electron and hole within the pair. To describe the MEL response on a phenomenological level, the following expressions were used:

$$\mathrm{MEL_S}(B) = \frac{P_\mathrm{S}(B)}{P_\mathrm{S}(0)} = 4 \cdot \left(P_\uparrow - P_\uparrow^2\right) - 4\alpha \frac{B^2}{\Delta B_{1/2}^2 + B^2} + d_\mathrm{S} \tag{7}$$

$$\mathrm{MEL_T}(B) = \frac{P_\mathrm{T}(B)}{P_\mathrm{T}(0)} = \frac{4}{3} \cdot \left(1 - P_\uparrow + P_\uparrow^2\right) + \frac{4\alpha}{3} \frac{B^2}{\Delta B_{1/2}^2 + B^2} + d_\mathrm{T}. \tag{8}$$

Here, d accounts for the effective contribution of low-field (hyperfine-mediated) effects that are relevant at fields below 800 mT. These two equations describe the measured MEL functionality at high magnetic fields well, but the parameters extracted should be viewed with caution because eq. (7) and (8) are merely phenomenological expressions. They are used mainly to estimate the relative contribution of TSP to the MEL data.

The fitted parameters for the MEL data shown in the main text are stated in Supplementary Tables 1-4.

Supplementary Note 3: Discussion of potential Joule heating effects

Joule heating effects are easily resolved in a departure from the theoretically predicted TSP effect, which occurs in low-quality devices at high operating currents (above 20 μA) and voltages (above 10 V). After optimizing of the device structure, we were able to reduce the heat dissipation of the OLED to ~3.5 μW for SYPPV OLEDs by exploiting improved charge-injection efficiency. The cooling power of the cryostat is approximately 1 mW. Even though it is hard to assess all of the relevant thermal conductivities, in particular the thermal transport through the glass substrate of the OLED, the fact that we observe almost perfect agreement of the temperature dependence of TSP for the SYPPV OLED with the set temperature of the cryostat implies that, for these very low operating powers, the device is, to all extents, in thermal equilibrium with the surroundings.

Not only the device performance plays an important role but also the cooling power of the cryostat. Both the helium gas flow through the sample chamber and the thermal coupling of the sample to the copper cold finger have a substantial impact on the cooling efficiency. Therefore, it is hard to estimate the effective cooling power available to the device and, consequently, to compare it to the Joule heating. One way to approach this problem is to compare the dynamic exchange-gas cooling method of the cryostat to direct cooling of the OLED by immersion in a liquid-helium bath. For all measurements shown in the main text, the devices were cooled by an evaporated helium gas flowing through the sample chamber, enabling us to reach variable temperatures. However, our cryogenic system also allows us to reach fixed temperatures of 4.2 K and 1.5 K, where the sample is completely submerged in liquid helium, either at atmospheric pressure or under vacuum pumping. Under these conditions, the surface of the OLED is in direct contact with the liquid helium, so we can assume that Joule heating is completely compensated and the temperature of the organic semiconductor is equal to the surrounding helium bath. By comparing these measurements in the liquid bath with the more versatile gas-phase measurements, we confirmed that the cooling works as desired under driving conditions for the dual-emitter OLEDs comparable to those used in the main text. For the temperature series of DMDB-PZ in the main text the Joule heating power was roughly 120 μW.

Supplementary Figures

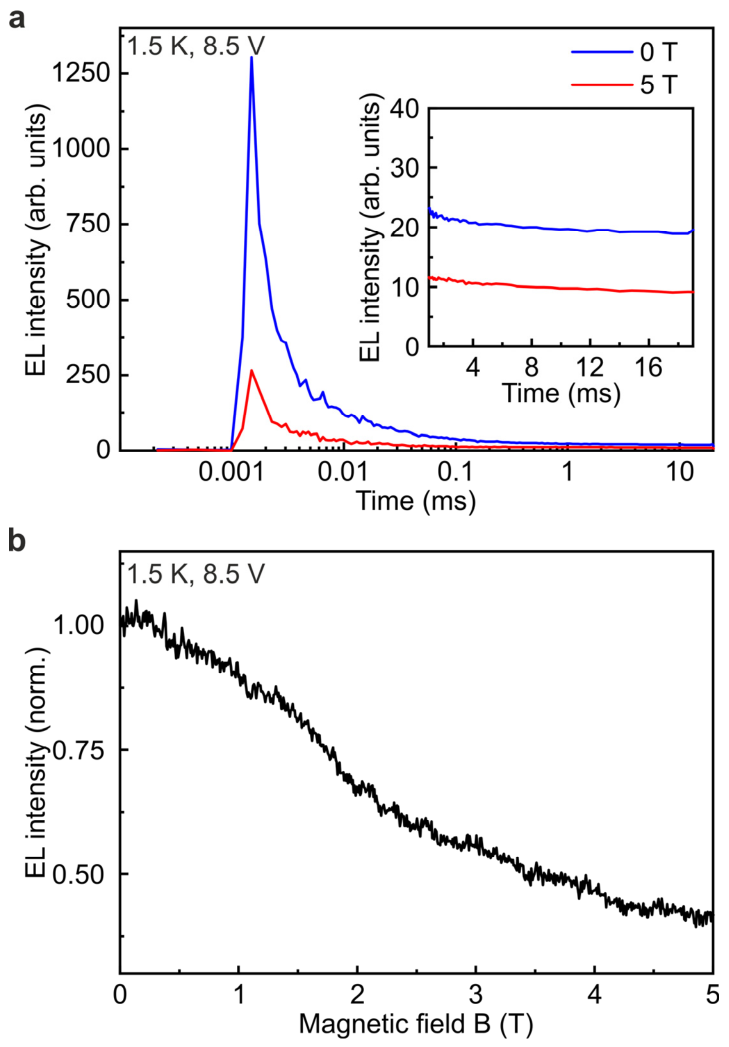

Supplementary Figure 1. Impact of TSP on the transient EL intensity at turn-on of a SYPPV OLED. a) The EL intensity is dominated by an overshoot in the first 10 μs of the voltage pulse. In contrast to the report of Wang *et al.*,[1] we find no indication in the data of triplet-triplet annihilation occurring. The EL intensity is quenched immediately at the pulse onset by TSP at high fields, implying that spin relaxation takes place prior to electron-hole recombination. It is therefore not possible to extract information on the spin relaxation time from the transient data. After reaching equilibrium in the EL intensity, shown in the inset, the transient EL is suppressed by the magnetic field by the same amount as in the static MEL measurements at this voltage, shown in b).

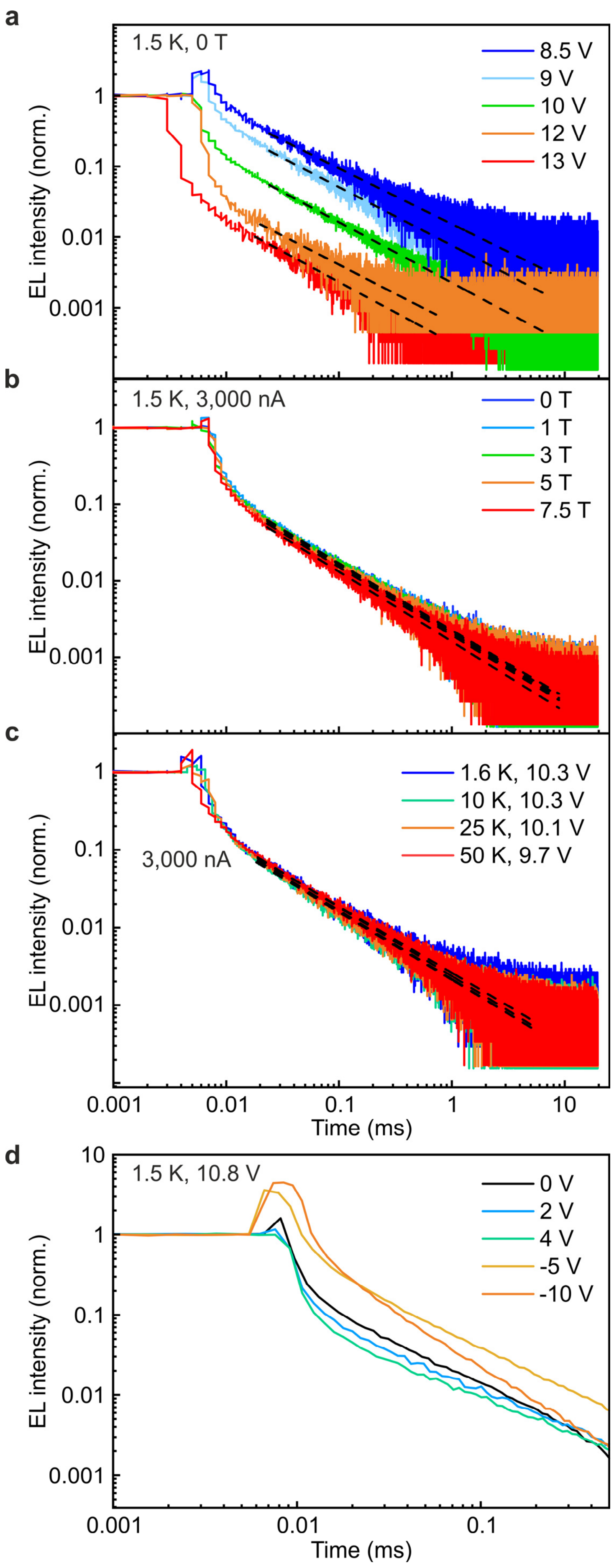

a
1.5 K, 0 T
8.5 V
9 V
10 V
12 V
13 V
EL intensity (norm.)
1
0.1
0.01
0.001
b
1.5 K, 3,000 nA
0 T
1 T
3 T
5 T
7.5 T
EL intensity (norm.)
c
3,000 nA
1.6 K, 10.3 V
10 K, 10.3 V
25 K, 10.1 V
50 K, 9.7 V
EL intensity (norm.)
0.001
0.01
0.1
1
10
Time (ms)
d
1.5 K, 10.8 V
0 V
2 V
4 V
-5 V
-10 V
EL intensity (norm.)
10
Time (ms)

Supplementary Figure 2. Transient EL intensity of a SYPPV OLED at turn-off of a voltage pulse. All data are normalized to the corresponding steady-state EL intensity. After a sharp EL overshoot, which is caused mainly by trapped charges, a power-law decay of the EL intensity is seen. Dashed lines indicate a power-law functionality of the form $t^{-\alpha}$, with α between 0.7 and 0.9.[2] a) Dependence of the decay dynamics on pulse voltage at zero field and 1.5 K. b) Dependence of the decay dynamics on magnetic field, following a pulse driving the OLED at a constant current of 3000 nA at 1.5 K. c) Dependence of the decay dynamics on temperature, following a pulse driving the OLED at a constant current of 3000 nA at zero field. Since the temperature slightly affects the resistance of the device, the pulse voltage is adjusted to reach the equilibrium current of 3000 nA. d) Dependence of the EL overshoot and decay dynamics on bias offset of the turn-on pulse. The dynamics are evidently insensitive to temperature and magnetic-field strength, implying that the transient EL at turn-off is not associated with delayed fluorescence from triplet-triplet annihilation but instead arises solely from trapped charges.

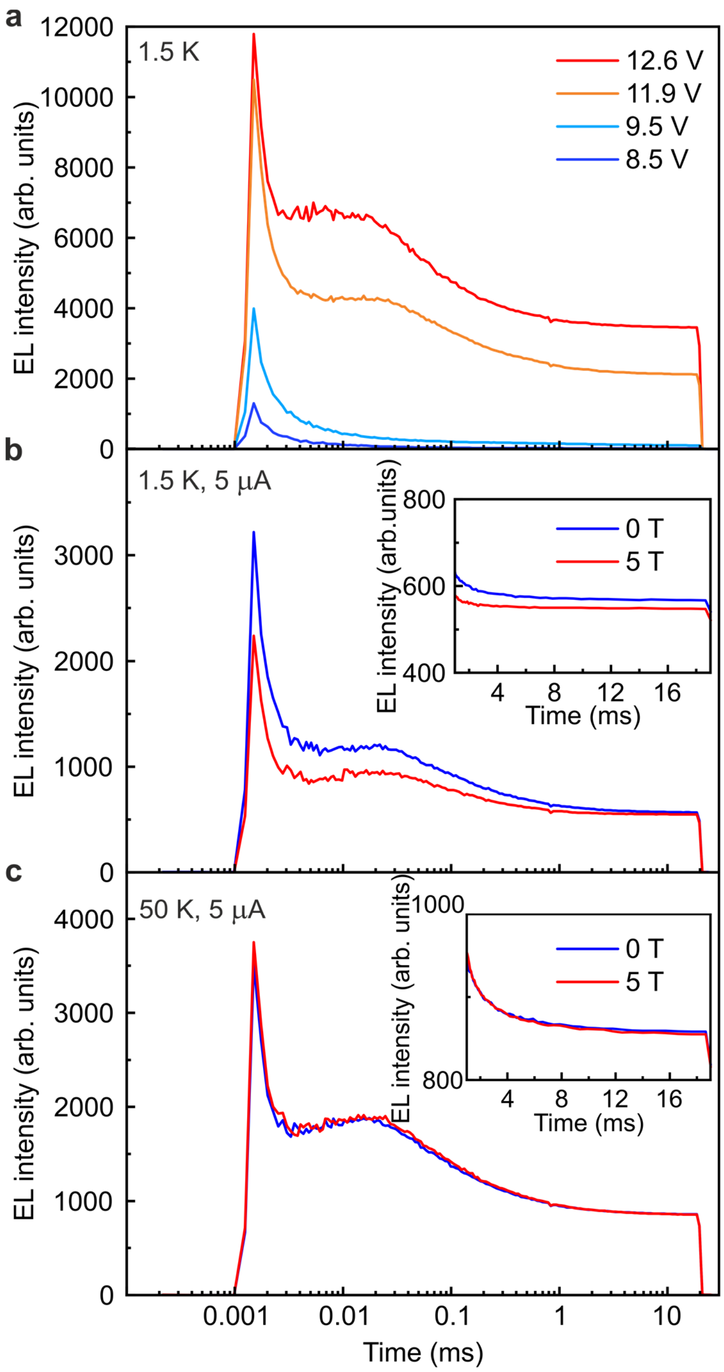

Supplementary Figure 3. Transient EL intensity of a SYPPV OLED over the entire voltage pulse of 20 ms duration. a) Voltage dependence. At high voltages an additional plateau in the EL intensity is seen. The sharp initial EL overshoot appears to be reduced at higher voltages, and is thus presumably linked to charge accumulation at internal energy barriers within the device. After approximately 1 ms, steady-state conditions are reached. b) Magnetic-field dependence at 1.5 K. After reaching the steady-state EL intensity, illustrated in the inset, the EL is reduced by a total of 5 % at 5 T due to TSP. However, suppression at this field of the initial overshoot and the plateau is much stronger. c) Magnetic-field dependence at

50 K. The magnetic field has no impact on the turn-on behaviour and on the steady-state EL intensity plotted in the inset, which is consistent with the absence of TSP at this temperature. The voltage of the pulse was chosen such that it corresponds to a steady-state device current of 5 μA.

Supplementary Tables

panel a	1.5 K	2.5 K	4.2 K	10 K	22 K
τ_c/τ_s	3.70	3.70	3.70	3.70	3.70

panel b	550 nA	800 nA	1000 nA	3000 nA
τ_c/τ_s	5.56	2.10	1.00	0.37

Supplementary Table 1. Fit parameters for the SYPPV OLED dataset in Figure 2a,b. Parameters for temperature a) and current dependencies b) were obtained by fitting with eq. (5).

left panel	Fluorescence (S)	Phosphorescence (T)
τ_c/τ_s	4.19	4.00

right panel	Fluorescence (S)	Phosphorescence (T)
τ_c/τ_s	0.98	0.90
$\Delta B_{1/2}$ (mT)	2.37	4.13
α	0.16	0.20
d	0.00	0.02

Supplementary Table 2. Fit parameters for Figure 3. Parameters in a) were obtained by using eq. (5) and (6). In b), the modified fit functions [eq. (7) and (8)] were used.

panel a:

**Fluorescence (S)**	2 µA	10 µA	100 µA
τ_c/τ_s	1.003	0.569	0.330
$\Delta B_{1/2}$ (mT)	52.97	7.271	7.198
α	0.950	0.086	0.089
d	-0.025	-0.005	-0.029

panel b:

**Phosphorescence (T)**	2µA	10 µA	100 µA
τ_c/τ_s	0.725	0.602	0.386
$\Delta B_{1/2}$ (mT)	60.310	31.140	8.969
α	0.985	0.363	0.036
d	0.033	0.019	-0.002

Supplementary Table 3. Fit parameters for the MEL current dependency of DMDB-PZ in Figure 4a,b. Parameters were extracted by fitting eq. (7) and (8).

panel c:

**Fluorescence (S)**	1.5 K	5 K	10 K
τ_c/τ_s	0.569	1.259	0.450
$\Delta B_{1/2}$ (mT)	7.271	8.154	31.440
α	0.086	0.019	0.283
d	-0.005	0.050	-0.011

panel d:

**Phosphorescence (T)**	1.5 K	5 K	10 K
τ_c/τ_s	0.602	0.570	0.612
$\Delta B_{1/2}$ (mT)	31.140	2.423	4.853
α	0.363	0.015	0.005
d	0.019	0.014	0.013

Supplementary Table 4. Fit parameters for the MEL temperature dependency of DMDB-PZ in Figure 4c,d. Parameters were extracted by fitting eq. (7) and (8).

Supplementary References

1 Wang, J. P., Chepelianskii, A., Gao, F. & Greenham, N. C. Control of exciton spin statistics through spin polarization in organic optoelectronic devices. *Nat. Commun.* **3**, 1191 (2012).

2 Reufer, M. *et al.* Spin-conserving carrier recombination in conjugated polymers. *Nat. Mater.* **4**, 340-346 (2005).

3 Kraus, H. *et al.* Visualizing the radical-pair mechanism of molecular magnetic field effects by magnetic resonance induced electrofluorescence to electrophosphorescence interconversion. *Phys. Rev. B* **95**, 241201 (2017).
